# Predictors of the risk of cognitive deficiency in very preterm infants: the EPIPAGE prospective cohort

**DOI:** 10.1111/j.1651-2227.2010.02064.x

**Published:** 2011-03

**Authors:** Ghada Beaino, Babak Khoshnood, Monique Kaminski, Stéphane Marret, Véronique Pierrat, Rachel Vieux, Gérard Thiriez, Jacqueline Matis, Jean-Charles Picaud, Jean-Christophe Rozé, Corine Alberge, Béatrice Larroque, Gérard Bréart, Pierre-Yves Ancel, for the EPIPAGE Study Group

**Affiliations:** 1 INSERM, UMR S953, Epidemiological Research Unit on Perinatal Health and Women's and Children's Health, Hôpital TenonF-75020, Paris, France; 2 Université Pierre et Marie CurieParis, 06, UMR S 953, F-75005, Paris, France; 3 INSERM, UMR S953, Epidemiological Research Unit on Perinatal Health and Women's and Children's Health, Hôpital CochinF-75014, Paris, France; 4 INSERM, UMR S953, Epidemiological Research Unit on Perinatal Health and Women's and Children's HealthVillejuif, F-94807, France; 5 Department of Neonatal Medicine, Rouen University Hospital, and the INSERM Avenir Research Group, Institute for Biomedical Research, University of RouenRouen, France; 6 Department of Neonatology, Jeanne de Flandres HospitalLille, France; 7 Maternite Regionale, Neonatal Department, Nancy-UniversityNancy, France; 8 Paediatric Intensive Care Unit, Saint Jacques HospitalBesançon, France; 9 Department of Neonatology, Strasbourg University HospitalStrasbourg, France; 10 Neonatology Department, University Hospital of MontpellierMontpellier, France; 11 Department of Neonatology, Children's HospitalNantes, France; 12 Neonatology Unit, Children's HospitalToulouse, France

**Keywords:** Cognitive deficiency, Predictor, Very preterm infant

## Abstract

**Aim:**

To assess cerebral lesions and other medical as well as social characteristics as predictors of risk of mild and severe cognitive deficiencies in very preterm infants.

**Methods:**

As part of the EPIPAGE population-based prospective cohort study, perinatal data and cognitive outcome at 5 years of age were recorded for 1503 infants born before 33 weeks of gestation in nine regions of France in 1997. Mild cognitive deficiency was defined as a Mental Processing Composite score on the Kaufman Assessment Battery for Children test of between 70 and 84, and severe cognitive deficiency as a score of <70.

**Results:**

After controlling for cerebral lesions and other medical as well as social factors, low parental socio-economic status and lack of breastfeeding were significant predictors of mild and severe cognitive deficiencies, whereas presence of cerebral lesions, being small for gestational age and having a large number of siblings were predictors of severe cognitive deficiency.

**Conclusion:**

Predictors of poor cognitive outcome in very preterm infants are low social status, lack of breastfeeding, presence of cerebral lesions on ultrasound scan, being born small for gestational age and having a high number of siblings. Social factors predicted both mild and severe cognitive deficiencies, whereas medical factors predicted mostly severe cognitive deficiencies.

## Introduction

Very preterm infants (born before 33 weeks of gestation) are at high risk of cognitive deficiency (1), which in turn is predictive of further learning difficulties and poor academic achievement (2,3). Recent large cohort studies have shown a rate of cognitive deficiencies in very preterm infants of around 30%, which is much higher than that of motor deficiencies in this group of children (around 10%) (4).

Cognitive deficiency in very preterm infants is a complicated multifactorial issue. It depends on both biological and environmental events. Biological events can include the formation of brain abnormalities that represent a combination of destructive and developmental mechanisms influenced by antenatal and postnatal factors (5). Environmental events may include a variety of processes that occur in childhood; for instance, children's emotional environment and the socio-economic context of their home, school and other places of social interactions.

Studies have identified several medical and social risk factors for cognitive deficiency. These factors include cerebral lesions, chronic lung disease, male sex and small head circumference, as well as ethnicity, parental occupation, maternal education and marital status (6–11). Yet, these studies had a number of limitations: small numbers of infants were followed up, few medical factors were studied, and social factors were not always evaluated together with medical factors. Moreover, none of these studies has considered mild versus moderate to severe cognitive deficiencies independently. Indeed, mild, moderate and severe cognitive outcomes are a continuum; however, risk factors can alter cognitive function to different extents.

In this study, we assessed cerebral lesions on neonatal cranial ultrasound scan and other medical and social characteristics as predictors of mild and severe cognitive deficiencies in a large population-based cohort of very preterm infants.

## Methods

This study was approved by the French data protection agency (Commission nationale de l'informatique et des libertés).

### Participants

**Figure 1:**
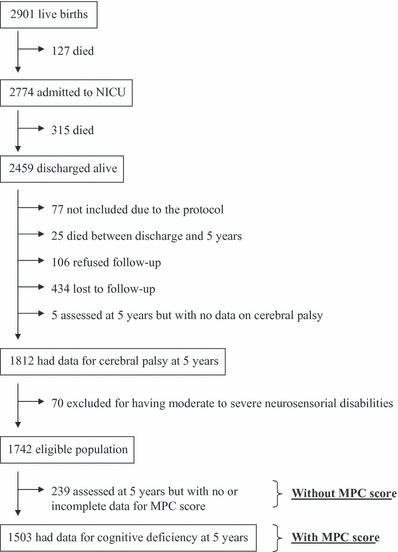
Study population.

All infants born between 22 and 32 weeks of gestation in nine regions of France throughout 1997 (2901 live births) were included in the EPIPAGE study (12) (Fig. 1). Of these infants, 2459 were discharged alive. The protocol included the option of following at random only one of every two infants born at 32 weeks of gestation, to reduce the regional workload. Two regions exercised this option and, as a consequence, 77 infants were not included in the follow-up. At hospital, parents were asked to give their informed consent for follow-up and were free to do so or to refuse without giving any justification. After exclusion of infants who died between hospital discharge and 5 years of age, those whose parents refused follow-up and those who were lost to follow-up, information on cerebral palsy at 5 years of age was available for 1812 infants (13). Furthermore, 70 infants were excluded for having moderate to severe neurosensorial disabilities defined as walking with aid or unable to walk or having severe hearing or visual deficiency (12), as infants with these disabilities may not be correctly evaluated for cognitive ability. Thus, 1742 infants constituted the eligible population.

### Five-year assessment

At 5 years, children were invited for a check-up organized for the study in every region and were assessed by trained psychologists blinded to their perinatal data. The assessment used the Kaufman Assessment Battery for Children (K-ABC) test, which was validated in France in 1990–1991 (14). Overall cognitive ability was evaluated by the Mental Processing Composite (MPC) score (mean ± SD: 100 ± 15), which was available for 1503 infants (86% of the eligible population). The remaining 239 infants who were assessed at 5 years of age but were not assigned an MPC score because of missing or incomplete test data differed from infants with a score by having more severe cerebral lesions, higher birth weight and lower parental socio-economic status (see Appendix for details).

Cognitive deficiency was classified as mild when the MPC score was between 70 and 84, and as moderate to severe (later simply classified as severe) when the MPC score was below 70.

### Medical characteristics in the perinatal period

The major characteristics recorded were cerebral lesions on neonatal cranial ultrasound scan, which is standard practice in very preterm infants. In France, usually one to three scans are performed during the first 2 weeks of life, then one every week for infants with lesions and one every 2 weeks for those without lesions (15). In total, 97% of the EPIPAGE study infants had at least one cranial ultrasound scan in the neonatal period; among these infants, 11% had one ultrasound scan, 23% had two and 66% had three or more scans (16). Two major types of cerebral lesion were assessed: intraventricular haemorrhage (IVH) and white matter disease [comprising intraparenchymal haemorrhage (IPH), periventricular leukomalacia (PVL) and ventricular dilatation] (17). Subependymal IVH was classified as grade I, intraventricular IVH as grade II and IVH associated with ventricular dilatation as grade III. IPH included large unilateral parenchymal hyperdensity or a large unilateral porencephalic cyst. PVL was defined as the presence of periventricular white matter echolucencies (cystic PVL) or echodensities persisting for more than 14 days without cyst formation. Ventricular dilatation was defined as an isolated dilatation of ventricles with no associated IVH. When several cerebral lesions were observed, the most severe lesion was considered; in order of decreasing severity: cystic PVL or IPH (class 1), persistent echodensities or ventricular dilatation or grade III IVH (class 2), grade II IVH (class 3) and grade I IVH (class 4). Infants without an identified cerebral abnormality constituted the reference group.

Other recorded medical characteristics included information on pregnancy, infant characteristics and postnatal factors. Information on pregnancy included single or multiple pregnancy; antenatal corticosteroid administration; and complications of pregnancy, classified according to the following priority order of maternal hypertension (systolic blood pressure ≥140 mmHg or diastolic blood pressure ≥90 mmHg during pregnancy), then antepartum haemorrhage, and then preterm premature rupture of membranes at least 12 h before the beginning of labour or idiopathic preterm labour (spontaneous onset of labour before rupture of membranes). Infant characteristics were the following: gestational age, in completed weeks of amenorrhoea (based on the date of the last menstrual period and an early prenatal ultrasound scan, which is standard practice for pregnant women in France (18)); infant sex; and whether the infant was small for gestational age (birth weight below the 10th centile of birth weight of live births stratified by week of gestational age and by gender in our population). Postnatal data included Apgar score at 1 min; respiratory distress syndrome; necrotizing enterocolitis; maternal–foetal infection (maternal-acquired culture-proven neonatal sepsis); bronchopulmonary dysplasia at 36-week gestational age (need of oxygen and/or breathing assistance); acute anaemia (haemoglobin <13 g/dL following perinatal haemorrhage); late-onset anaemia; postnatal corticosteroid administration; and breastfeeding at hospital discharge (defined as receiving exclusive or mixed breast milk feeds at discharge of the infant from hospital).

### Social characteristics

Social characteristics considered were parental socio-economic status; defined as the highest occupational status between the mother and the father and classified into high (professional), high intermediate (intermediate, administrative/public service, self-employed or student), low intermediate (shop assistant or service worker) and low (manual worker or unemployed); maternal educational level, classified into postsecondary education, high school level and less or no education; number of siblings in the family; and maternal age at birth. Information on parental socio-economic status and maternal educational level was obtained in the neonatal period or, when incomplete or unavailable, at the 5-year follow-up.

### Statistical analysis

We assessed medical and social characteristics in very preterm infants as potential predictors of cognitive deficiencies but did not aim to elucidate causal pathways or estimate causal effects. Specifically, two issues were considered, mild cognitive deficiency and severe cognitive deficiency, and predictors of each were studied in univariate then in multivariate modelling.

First, univariate multinomial logistic regression was used to identify medical and social characteristics as risk factors separately for mild and severe cognitive deficiency. Second, a multivariate multinomial logistic regression model was used to assess independent predictors of mild versus severe cognitive deficiency among medical and social characteristics. Predictor variables that were studied in multivariable analyses were chosen based on previous studies (6–12) and the results of our univariate analyses. The medical factors were neonatal cerebral lesions, gestational age of 28 weeks or less, infant sex, small for gestational age, Apgar score at 1 min below 7, necrotizing enterocolitis, bronchopulmonary dysplasia at 36 weeks, acute anaemia, late-onset anaemia and postnatal corticosteroid. The social factors included parental socio-economic status and number of siblings. The variable breastfeeding reflected both medical and social contexts as it is not only an indicator of infant nutrition but also an indirect social indicator that reflects the mother's behaviour. The rationale for entering parents’ socio-economic status rather than mother's educational level was to consider both parents in assessing social status; in addition, more complete data were available in our study for parents’ socio-economic status than for mother's educational level. Predictors of mild and severe cognitive deficiency were compared using the Wald test assessing heterogeneity in odds ratios for each predictor in the multinomial logistic model.

Results were expressed as crude and adjusted odds ratios and their 95% confidence interval (CI). Complementary analysis was conducted after excluding children with aid-free ambulatory cerebral palsy (walking alone) to assess any change in predictors of cognitive outcome that may be influenced by mild motor deficiency.

Statistical analyses were performed using STATA/SE, version 10, (StataCorp, College Station, TX, USA).

## Results

We found that the prevalence of mild cognitive deficiency for children without moderate to severe neurosensorial disabilities was 21% (95% CI: 19–23%) and that of severe cognitive deficiency was 11% (95% CI: 10–13%). In this same population, mean MPC score was 94 (SD 19).

In univariate analysis, characteristics significantly associated with mild cognitive deficiency were bronchopulmonary dysplasia at 36 weeks, no breastfeeding, low parental socio-economic status, low level of maternal education and large number of siblings (Table 1). Characteristics associated with severe cognitive deficiency included those associated with mild deficiency as well as the presence of cerebral lesions, lower gestational age, small for gestational age, Apgar score at 1 min below 7, anaemia and postnatal corticosteroid administration (Table 1).

**Table 1:** Crude associations between medical and social characteristics and mild and severe cognitive deficiencies

		Mild cognitive deficiency		Severe cognitive deficiency	
					
	No.	%	Odds ratio‡(95% CI)	%	Odds ratio†(95% CI)
Medical characteristics					
Gestational age (weeks)					
31–32	729	20	1.00	9	1.00*
29–30	409	25	1.36 (1.01–1.82)	9	1.15 (0.75–1.76)
27–28	263	17	0.96 (0.66–1.40)	19	2.42 (1.61–3.64)
24–26	102	23	1.39 (0.84–2.30)	16	2.10 (1.14–3.85)
Infant sex					
Female	733	22	1.00	10	1.00
Male	770	20	0.95 (0.74–1.22)	12	1.22 (0.88–1.70)
Small for gestational age§					
No	1364	21	1.00	10	1.00*
Yes	133	22	1.19 (0.76–1.86)	18	1.96 (1.20–3.20)
Multiple pregnancy					
No	1037	20	1.00	12	1.00
Yes	466	23	1.19 (0.91–1.55)	10	0.91 (0.64–1.31)
Complications of pregnancy					
Maternal hypertension	439	22	1.00	14	1.00
Antepartum haemorrhage	140	23	1.02 (0.64–1.62)	13	0.94 (0.52–1.67)
Preterm premature rupture of membranes or preterm labour	834	20	0.84 (0.63–1.12)	10	0.64 (0.45–0.92)
Other complications	83	16	0.61 (0.32–1.16)	11	0.69 (0.32–1.47)
Antenatal corticosteroids					
No	358	21	1.00	13	1.00
Yes	1120	21	0.95 (0.71–1.29)	11	0.78 (0.54–1.13)
Cerebral lesion					
None	991	20	1.00	8	1.00*
Grade I IVH	146	23	1.22 (0.79–1.86)	12	1.53 (0.87–2.69)
Grade II IVH	96	21	1.18 (0.69–2.01)	17	2.30 (1.27–4.19)
Grade III IVH or persistent echodensities or ventricular dilatation	221	23	1.41 (0.98–2.02)	19	2.84 (1.87–4.30)
Cystic PVL or IPH	30	23	1.91 (0.75–4.85)	33	6.65 (2.83–15.65)
Apgar score at 1 min <7					
No	839	20	1.00	10	1.00*
Yes	578	22	1.17 (0.90–1.52)	13	1.47 (1.05–2.06)
Respiratory distress syndrome					
No	851	21	1.00	11	1.00
Yes	643	21	1.03 (0.79–1.33)	12	1.10 (0.79–1.52)
Necrotizing enterocolitis					
No	1436	21	1.00	11	1.00
Yes	52	25	1.36 (0.70–2.64)	15	1.57 (0.71–3.49)
Maternal–foetal infection					
No	1375	22	1.00	11	1.00
Yes	96	16	0.66 (0.37–1.18)	11	0.95 (0.49–1.83)
Bronchopulmonary dysplasia					
No	1290	21	1.00*	10	1.00*
Yes	176	26	1.52 (1.04–2.22)	18	2.16 (1.40–3.36)
Acute anaemia					
No	1394	21	1.00	11	1.00*
Yes	90	20	1.03 (0.59–1.78)	18	1.79 (1.00–3.20)
Late-onset anaemia					
No	890	20	1.00	8	1.00*
Yes	589	22	1.24 (0.96–1.61)	16	2.27 (1.63–3.17)
Postnatal corticosteroids					
No	1228	21	1.00	10	1.00*
Yes	266	22	1.20 (0.86–1.67)	18	2.05 (1.41–2.99)
Breastfeeding					
No	1109	23	1.00*	13	1.00*
Yes	312	15	0.54 (0.39–0.76)	4	0.25 (0.14–0.44)
Social characteristics					
Parents' socio-economic status¶					
High	242	14	1.00*	7	1.00*
High intermediate	760	18	1.39 (0.92–2.10)	10	1.59 (0.90–2.80)
Low intermediate	219	25	2.39 (1.47–3.87)	16	3.14 (1.66–5.92)
Low	277	32	3.45 (2.20–5.42)	16	3.75 (2.04–6.90)
Mother's educational level					
Postsecondary education	481	14	1.00*	6	1.00*
High school level	320	15	1.13 (0.75–1.69)	7	1.14 (0.65–2.01)
Less or no education	674	28	2.90 (2.12–3.96)	17	3.71 (2.43–5.66)
Maternal age at birth (years)					
<25	293	21	1.00	12	1.00
25–29	551	20	0.91 (0.64–1.31)	11	0.90 (0.57–1.41)
30–34	418	20	0.88 (0.61–1.28)	8	0.61 (0.37–1.02)
≥35	234	24	1.30 (0.85–1.97)	16	1.53 (0.92–2.54)
Number of siblings					
0	838	19	1.00*	9	1.00*
1–2	523	23	1.33 (1.02–1.75)	12	1.52 (1.06–2.17)
≥3	135	29	2.25 (1.46–3.46)	21	3.50 (2.13–5.75)

PVL = periventricular leukomalacia; IPH = intraparenchymal haemorrhage; IVH = intraventricular haemorrhage.

No. is the number of children with data on Mental Processing Composite (MPC) score for each category of characteristic; % is the proportion of children with mild or severe cognitive deficiency relative to the number of children for each category of characteristic.

* p < 0.05.

† Mild cognitive deficiency (MPC 70–85) compared to no cognitive deficiency (MPC ≥ 85).

‡ Severe cognitive deficiency (MPC < 70) compared to no cognitive deficiency (MPC ≥ 85).

§ Small for gestational age corresponds to birth weight below the 10th centile of birth weight of live births stratified by week of gestational age and by gender in our population.

¶ The parent's socio-economic status is defined as the highest occupational status between the mother's and the father's status and classified into high (professional), high intermediate (intermediate, administrative/public service, self-employed or student), low intermediate (shop assistant or service worker) and low (manual worker or unemployed).

In multivariate analysis, parents’ socio-economic status was the main predictor of mild cognitive deficiency (odds ratio for low socio-economic status versus high status: 3.43; 95% CI: 2.01–5.83) (Table 2). Bronchopulmonary dysplasia was associated with mild cognitive deficiency, although this association did not reach statistical significance (odds ratio: 1.57; 95% CI: 0.97–2.54) (Table 2). In addition, gestational age of 28 weeks or less was associated with lower odds of mild cognitive deficiency (odds ratio: 0.61; 95% CI: 0.40–0.93), as was breastfeeding (odds ratio: 0.66; 95% CI: 0.45–0.96) (Table 2). By contrast, predictors of severe cognitive deficiency were mainly cerebral lesions on ultrasound scan (odds ratio for cystic PVL or IPH versus no cerebral lesion: 6.37; 95% CI: 2.46–16.54), low parental socio-economic status (odds ratio for low versus high socio-economic status: 2.60; 95% CI: 1.29–5.24), high number of siblings (odds ratio for having three or more siblings versus no siblings: 2.84; 95% CI: 1.59–5.10) and being born small for gestational age (odds ratio: 2.49; 95% CI: 1.41–4.40) (Table 2). Breastfeeding was also highly associated with lower odds of severe cognitive deficiency (odds ratio: 0.32; 95% CI: 0.17–0.62). The difference in odds ratios for mild versus severe cognitive deficiency was not statistically significant for parents’ socio-economic status and bronchopulmonary dysplasia (p-value for heterogeneity in odds ratios = 0.4 and 0.2, respectively) but was almost significant for cerebral lesions and number of siblings (p-value for heterogeneity in odds ratios = 0.08 and 0.1, respectively) and significant for gestational age of 28 weeks or less and small for gestational age (p-value for heterogeneity in odds ratios = 0.01 and 0.007, respectively). Replacing parents’ socio-economic status by mothers’ educational level in multivariate analyses produced similar results.

**Table 2:** Multinomial logistic regression models analysing the association between medical and social risk factors and mild and severe cognitive deficiency: the EPIPAGE cohort study

	Mild cognitive deficiency	Severe cognitive deficiency	
			
Risk factor	Odds ratio (95% CI)	Odds ratio (95% CI)	p-value*
Cerebral lesion			
None	1.00	1.00	0.08
Grade I IVH	1.09 (0.67–1.76)	1.39 (0.74–2.60)	
Grade II IVH	1.15 (0.64–2.08)	1.88 (0.95–3.72)	
Grade III IVH or echodensities or ventricular dilatation	1.33 (0.87–2.04)	2.51 (1.53–4.11)	
Cystic PVL or IPH	1.98 (0.71–5.50)	6.37 (2.46–16.54)	
Gestational age ≤28 weeks			
No	1.00	1.00	0.01
Yes	0.61 (0.40–0.93)	1.28 (0.78–2.08)	
Infant sex			
Female	1.00	1.00	0.2
Male	0.80 (0.60–1.07)	1.08 (0.74–1.57)	
Small for gestational age			
No	1.00	1.00	0.007
Yes	1.01 (0.59–1.70)	2.49 (1.41–4.40)	
Apgar score at 1 min <7			
No	1.00	1.00	1.0
Yes	1.14 (0.85–1.54)	1.14 (0.77–1.69)	
Necrotizing enterocolitis			
No	1.00	1.00	0.4
Yes	1.33 (0.64–2.76)	0.84 (0.33–2.15)	
Bronchopulmonary dysplasia			
No	1.00	1.00	0.2
Yes	1.57 (0.97–2.54)	1.09 (0.62–1.90)	
Acute anaemia			
No	1.00	1.00	0.3
Yes	0.68 (0.33–1.39)	1.08 (0.53–2.19)	
Late-onset anaemia			
No	1.00	1.00	0.3
Yes	1.10 (0.78–1.55)	1.45 (0.93–2.25)	
Postnatal corticosteroid use			
No	1.00	1.00	0.6
Yes	1.33 (0.84–2.12)	1.14 (0.66–1.97)	
Breastfeeding			
No	1.00	1.00	0.05
Yes	0.66 (0.45–0.96)	0.32 (0.17–0.62)	
Parents' socio-economic status†			
High	1.00	1.00	0.4
High intermediate	1.42 (0.88–2.28)	1.23 (0.65–2.32)	
Low intermediate	2.19 (1.26–3.82)	2.89 (1.42–5.88)	
Low	3.43 (2.01–5.83)	2.60 (1.29–5.24)	
Number of siblings			
0	1.00	1.00	0.1
1–2	1.24 (0.91–1.69)	1.52 (1.01–2.30)	
≥3	1.39 (0.84–2.30)	2.84 (1.59–5.10)	

PVL = periventricular leukomalacia; IPH = intraparenchymal haemorrhage; IVH = intraventricular haemorrhage.

* p-value for heterogeneity of odds ratios for predictors of mild versus severe cognitive deficiency.

† The parent's socio-economic status is defined as the highest occupational status between the mother's and the father's status and classified into high (professional), high intermediate (intermediate, administrative/public service, self-employed or student), low intermediate (shop assistant or service worker) and low (manual worker or unemployed).

After exclusion of children with aid-free ambulatory cerebral palsy, the only notable change in the odds ratios was in the case of the association between severe cerebral lesions (cystic PVL or IPH) and severe cognitive deficiency for which the odds appeared to be lower (odds ratio: 3.83; 95% CI: 1.10–13.29), although still statistically significant.

## Discussion

EPIPAGE is the largest population-based cohort assessing the outcome of very preterm children at 5 years of age in a contemporary obstetric and neonatal care context. It is also the first study to assess risk factors for mild and severe cognitive deficiency considering a large set of medical and social characteristics. We found that mild cognitive deficiency was mainly influenced by social status and breastfeeding, whereas severe cognitive deficiency was mainly influenced by cerebral lesions, in addition to social status, breastfeeding, having a high number of siblings and being small for gestational age. In particular, after controlling for medical and social factors, the odds of severe cognitive deficiency were increased sixfold in infants with cystic PVL or IPH and twofold in those born to parents of low socio-economic status.

Cognitive ability in our study was assessed according to the MPC score, an IQ-equivalent measure that assesses overall cognitive ability of infants including sequential and simultaneous processing. MPC score is predictive of future learning difficulties and poor academic achievement (2,3). Excluding children with moderate to severe neurosensorial disabilities in our study allowed to prevent inaccurate estimation of cognitive outcome in this population as the test we used was not adapted to these disabilities. In addition, the parents of 106 infants refused follow-up, 434 infants were lost to follow-up, and 239 infants were assessed at 5 years of age but were not assigned an MPC score. These infants tended to be of lower socio-economic spheres (13) (see Appendix), which probably led to an under-estimation of the prevalence of cognitive deficiencies in our study. It is difficult to predict the effect of missing data on the relation between cognitive deficiency and the factors evaluated though there is no robust evidence for any bias in the associations.

In our study, cerebral lesions were associated with severe, but not mild, cognitive deficiency. To our knowledge, EPIPAGE is the first study to consider risk factors of mild versus severe deficiency, relying on the hypothesis that risk factors may alter cognitive function to different extents. However, diagnosis of cerebral abnormalities by ultrasonography was not optimal for two important reasons. First, in our population-based study, we obtained information on cerebral lesions based on routine practice. Therefore, there was no unique standardized protocol for cranial scanning follow-up, and staff qualifications were heterogeneous. Although during the study period cranial ultrasound was generally performed with high-frequency 7.5 MHz transducers by qualified neonatalogists or radiologists who routinely performed ultrasonography, interobserver reliability in interpreting cranial ultrasound scan has been shown to be relatively poor for low grade IVHs resulting in some probable misclassifications (19). No cerebral lesions were apparent on the neonatal cranial ultrasound scan in half of all children with severe cognitive deficiency in our study. Some of these infants may have had sonographic cerebral lesions that were missed. Use of an optimal ultrasound scanning protocol may have improved the accuracy of cerebral lesion diagnosis. Although magnetic resonance imaging is more sensitive than a cranial ultrasound scan, especially for detecting diffuse cranial abnormalities (20–22), it is not yet standard practice in very preterm infants because it is expensive and requires sedation and transport of infants. Second, it is now established that brain abnormality in the premature infant does not consist simply of destructive non-haemorrhagic and haemorrhagic lesions but indeed involves a more complex combination of destructive and developmental mechanisms, specifically impaired trophic/maturational processes (5). In this study, we could not explore neuronal/axonal disease, an under-recognized maturational disturbance that is diagnosed by volumetric and diffusion tensor magnetic resonance imaging and seems to relate strongly to cognitive deficits (5).

Social status is a major factor to be considered when assessing cognitive prognosis of very preterm infants. Three studies have used multivariate analysis to determine the associations between several social factors and the occurrence of cognitive deficiency, after controlling for medical factors (6–8). In the first of these studies, Taylor et al. (6) assessed a regional cohort of 68 very low birth weight infants at 5–9 years using the MPC score and showed that the neonatal risk index (including cerebral lesions and several other medical factors) was the most consistent predictor of outcome after controlling for age, gender and social risk factors. In the second study, Hack et al. prospectively assessed a centre-based cohort of 221 extremely low birth weight infants at 20 months corrected age. They found that social risk, defined according to maternal marital status, race and education, was a significant predictor of a Mental Development Index score of the Bayley Scales of Infant Development below 70 (7). The third more recent study included a regional prospective cohort of 151 very preterm infants assessed at 18 and 24 months corrected age. Ethnicity and maternal age at birth were found to be independent predictors for delayed mental development at 24 months corrected age but not at 18 months (8). In our population-based study, we assessed a much larger number (n = 1503) of very preterm infants. Cognitive outcome was evaluated at 5 years of age, and mild and severe cognitive deficiencies were studied. Our results confirmed the impact of low social status on cognitive outcome, even after controlling for cerebral lesions and numerous other medical factors.

In our large population-based study, cerebral lesions and being small for gestational age were significant predictors of mild and severe cognitive deficiencies after controlling for several other medical and social factors. This result is consistent with previous findings of an association between several medical factors and poor cognitive outcome at 5 years of age after adjustment for social characteristics (6). However, male sex was not an independent predictor of poor cognitive outcome in EPIPAGE, in contrast to the results of previous studies (7,8). Also unexpectedly, low gestational age was associated with lower odds of mild cognitive deficiency. In fact, non-responders to the MPC score born at 27–28 weeks of gestation had more cystic PVL and were less likely to be breastfed when compared to the non-responders of the other age groups. Knowing that children with cystic PVL are at high risk of neurosensorial disabilities and that absence of breastfeeding is a risk factor for mild but also for severe cognitive deficiencies, a selection bias due in part to the exclusion of children with neurosensorial disabilities could explain the lower odds of mild cognitive deficiency in infants born at 28 weeks and less. Moreover, adjustment for several other factors that are likely to be on the causal pathway between gestational age and outcome, especially cerebral lesions, could also explain this unexpected finding for gestational age.

We also explored breastfeeding, an infant factor that expresses both medical and social characteristics. A meta-analysis of 20 studies, published in 1999, concluded that breastfeeding has a beneficial effect on cognitive outcome (23). Recently, the beneficial effect of breastfeeding on the cognitive development of infants was confirmed through a large cluster-randomized trial of healthy term infants (n = 13889) evaluating a breastfeeding promotion intervention: the experimental group had a +5.9 cluster-adjusted mean difference for full-scale IQ (24). In the EPIPAGE population-based study, we also showed that breastfeeding at discharge of the infant from hospital in the specific population of very preterm infants was a significant predictor of lower risk of mild and severe cognitive deficiencies as assessed by the MPC score at the age of 5.

In conclusion, our findings point to the importance of medical characteristics for predicting severe cognitive deficiency in very preterm infants. After taking into account several medical and social characteristics, the presence of cerebral lesions and being small for gestational age were significant predictors of severe cognitive deficiency. Our study also underscores the extent to which social characteristics influence cognitive ability in very preterm infants. In particular, after taking into account cerebral lesions and several other medical characteristics, infants born to parents with low socio-economic status still had more than a twofold increase in the odds of severe cognitive deficiency. Of interest, our study showed that breastfeeding, a generally understudied infant characteristic, is highly associated with lower odds of cognitive deficiencies. Finally, predictors of cognitive deficiencies certainly give a better idea of the prognosis of infants; however, prediction of cognitive outcome at the individual level is still difficult to achieve (25).

## Abbreviations

CI: Confidence interval
IPH: Intraparenchymal haemorrhage
IVH: Intraventricular haemorrhage
K-ABC test: Kaufman assessment battery for children test
MPC score: Mental processing composite score
PVL: Periventricular leukomalacia

## Appendix

### EPIPAGE study group

*INSERM U953*: B Larroque (national coordinator), PY Ancel, B Blondel, G Bréart, M Dehan, M Garel, M Kaminski, F Maillard, C du Mazaubrun, P Missy, F Sehili, K Supernant; *Alsace*: M Durant, J Matis, J Messer, A Treisser (Hôpital de Hautepierre, Strasbourg); *Franche-comté*: A Burguet, L Abraham-Lerat, A Menget, P Roth, J-P Schaal, G Thiriez (CHU St Jacques, Besançon); *Haute-normandie*: C Lévêque, S Marret, L Marpeau (Hôpital Charles Nicolle, Rouen); *Languedoc-roussillon*: P Boulot, J-C Picaud (Hôpital Arnaud de Villeneuve, Montpellier), A-M Donadio, B Ledésert (ORS Montpellier); *Lorraine*: J Fresson, M André, JM Hascoët (Maternité Régionale, Nancy); *Midi-pyrénées*: C Arnaud, S Bourdet-Loubère, H Grandjean (INSERM U558, Toulouse), M Rolland (Hôpital des enfants, Toulouse); *Nord-pas-de-calais*: C Leignel, P Lequien, V Pierrat, F Puech, D Subtil, P Truffert (Hôpital Jeanne de Flandre, Lille); *Pays de la loire*: G Boog, V Rouger-Bureau, J-C Rozé (Hôpital Mère-Enfants, Nantes); *Paris-Petite-Couronne*: P-Y Ancel, G Bréart, M Kaminski, C du Mazaubrun (INSERM U149, Paris), M Dehan, V Zupan (Hôpital Antoine Béclère, Clamart), M Vodovar, M Voyer (Institut de Puériculture, Paris).

**Table:** Comparison of characteristics between infants assigned an MPC score at the 5-year assessment and those with no score

Characteristic	With MPC score n = *1503* No. (%)	Without MPC score n = *239* No. (%)	p-value
Cerebral lesion	*1484*	*235*	0.001
Cystic PVL	(2)	(5)	
IPH	(0)	(2)	
Persistent echodensities or ventricular dilatation	(13)	(14)	
Grade III IVH	(2)	(1)	
Grade II IVH	(6)	(7)	
Grade I IVH	(10)	(9)	
None	(67)	(62)	
Gestational age at birth (weeks)	*1503*	*239*	0.07
24–28	(24)	(24)	
29–30	(27)	(20)	
31–32	(48)	(55)	
Birth weight (g)	*1497* (1361 ± 387*)	*236* (1402 ± 429*)	0.1
Male sex	*1503* (51)	*239* (53)	0.6
Multiple pregnancy	*1503* (31)	*239* (33)	0.4
Parents' socio-economic status†	*1498*	*235*	<0.001
High	(16)	(9)	
High intermediate	(51)	(41)	
Low intermediate	(15)	(17)	
Low	(18)	(33)	

PVL = periventricular leukomalacia; IPH = intraparenchymal haemorrhage; IVH = intraventricular haemorrhage.

Numbers in italic are denominators for each characteristic.

* Values are means ± SD.

† The parent's socio-economic status is defined as the highest occupational status between the mother's and the father's status and classified into high (professional), high intermediate (intermediate, administrative/public service, self-employed or student), low intermediate (shop assistant or service worker) and low (manual worker or unemployed).
